# Does Metal Transfer Differ on Retrieved Ceramic and CoCr Femoral Heads?

**DOI:** 10.1155/2015/283038

**Published:** 2015-10-25

**Authors:** Eliza K. Fredette, Daniel W. MacDonald, Richard J. Underwood, Antonia F. Chen, Michael A. Mont, Gwo-Chin Lee, Gregg R. Klein, Clare M. Rimnac, Steven M. Kurtz

**Affiliations:** ^1^Implant Research Center, School of Biomedical Engineering, Science and Health Systems, Drexel University, Philadelphia, PA 19104, USA; ^2^Exponent, Inc., Philadelphia, PA 19104, USA; ^3^Rothman Institute at Thomas Jefferson University Hospital, Philadelphia, PA 19107, USA; ^4^Center for Joint Preservation and Replacement, The Rubin Institute of Advanced Orthopedics, Sinai Hospital of Baltimore, Baltimore, MD 21215, USA; ^5^The University of Pennsylvania, Philadelphia, PA 19104, USA; ^6^Hartzband Center for Hip and Knee Replacement, Paramus, NJ 07652, USA; ^7^Center for the Evaluation of Implant Performance, Departments of Orthopaedics and Mechanical and Aerospace Engineering, Case Western Reserve University, Cleveland, OH 44106, USA

## Abstract

Metal transfer has been observed on retrieved THA femoral heads for both CoCr and ceramic bearing materials. *In vitro* wear testing has shown increased wear to polyethylene acetabular liners with the presence of metal transfer. This study sought to investigate the extent of metal transfer on the bearing surface of CoCr and ceramic femoral heads and identify prevalent morphologies. Three bearing couple cohorts: M-PE (*n* = 50), C-PE (*n* = 35), and C-C (*n* = 15), were derived from two previously matched collections (*n* = 50/group) of CoCr and ceramic femoral heads. From the three cohorts, 75% of the femoral heads showed visual evidence of metal transfer. These femoral heads were analyzed using direct measurement, digital photogrammetry, and white light interferometry. Surface area coverage and curved median surface area were similar among the three cohorts. The most prevalent metal transfer patterns observed were random stripes (*n* = 21/75), longitudinal stripes (*n* = 17/75), and random patches (*n* = 13/75). Metal transfer arc length was shorter in the M-PE cohort. Understanding the morphology of metal transfer may be useful for more realistic recreation of metal transfer in *in vitro* pin-on-disk and joint simulators studies.

## 1. Introduction

Metal transfer has been observed on the femoral head components of revised total hip replacements for decades, appearing dark and metallic in color [1, 2]. The mechanisms of metal transfer to the bearing surface are thought to include femoral head dislocation, closed reduction procedures, impingement, or third body entrapment in the articulating zone [3–5]. Long longitudinal stripes have been observed when the femoral head and metal shell come into contact, referred to in past reports as “longitudinal scraping” on the bearing surface with both metal transfer and femoral head material loss [3, 6]. Additionally, debris entrapped in the counterface has been linked to patterned markings on the bearing surface [5, 7]. Metal transfer markings may consist of titanium (Ti) or cobalt chromium (CoCr) alloy and have been shown to increase the surface roughness of both ceramic and CoCr femoral heads [1, 8]. Metal transfer is a concern because some studies have correlated this increased surface roughness of the femoral head to an increased wear rate of the (conventional, i.e., not highly cross-linked) polyethylene counterface [8, 9].

Little is known about the morphology of metal transfer on ceramic or CoCr femoral heads or whether clinical and material factors influence either the extent or the morphology of metal transfer. Previous studies of metal transfer consisted of visual observation, semiquantitative scoring, and surface roughness measurements but failed to distinguish among the different morphologies of the metal transfer observed [1, 3, 8]. A recent set of case studies with six analyzed components described global patterns observed on the bearing surface of CoCr femoral heads for revised total hip replacements, but the patterns noted were not specific to metal transfer [7]. Recent studies have also established methods to ascertain global positions of wear patterns on CoCr femoral heads for computer-generated models simulating conventional polyethylene wear and liner positioning [6, 9]. These studies focused only on scratches and material removal of CoCr femoral heads, disregarding ceramic heads, alternative bearings, and metal transfer onto the bearing surface. To our knowledge, metal transfer evaluation has yet to be performed on ceramic femoral heads or on a large cohort of retrieved implants.

In this study, we asked the following questions: (1) what is the extent of metal transfer on the bearing surface of ceramic and CoCr femoral heads, and (2) does one bearing couple have a higher incidence of metal transfer? Additionally, we asked: (3) is there a specific pattern and morphology of metal transfer that occurs most often, and (4) does the metal transfer pattern or morphology differ between ceramic and CoCr femoral heads?

## 2. Methods

### 2.1. Cohort Selection

From 2001 to 2014, over 3,000 total hip replacement systems were collected at revision surgery as part of a multi-institutional, institutional review board-approved orthopaedic implant retrieval program, including six medical centers and three biomedical engineering laboratories. We utilized two previously matched groups of CoCr and ceramic heads. The CoCr femoral heads articulated with conventional polyethylene (*n* = 11) and HXLPE (*n* = 39). The ceramic femoral heads were a mix of alumina on alumina (*n* = 15), alumina on conventional polyethylene (*n* = 12), alumina on highly cross-linked polyethylene (HXLPE) (*n* = 8), zirconia-toughened-alumina (ZTA) on conventional polyethylene (*n* = 3), and zirconia-toughened-alumina (ZTA) on HXLPE (*n* = 12). The bearing couples were grouped into three cohorts overall: CoCr-on-polyethylene (M-PE), ceramic-on-polyethylene (C-PE), and ceramic-on-ceramic (C-C).

The M-PE components were implanted for a median of 1.9 years (Interquartile Range [IQR]: 2.7 years), the C-PE components for 2.1 years (IQR: 4.4 years), and the C-C components for 1.7 years (IQR: 1.4 years; *p* = 0.77). Although not specifically matched for, head size was similar among the three cohorts with a median head size of 32 mm (*p* = 0.47). Components in all cohorts were revised primarily for loosening and infection. Only one implant from the C-PE (ZTA on HXLE) cohort was revised for instability. Gender, body mass index (BMI), University of California Los Angeles activity score (UCLA), and implantation time were similar among all cohorts (*p* > 0.05; Table 1). The C-PE and C-C cohorts were slightly younger than the M-PE cohort (mean difference = 3 and 10 years, resp.; *p* = 0.02; Table 1).

All components were cleaned using the same institutional procedure. Specifically, the CoCr and ceramic femoral heads were individually soaked in 10 : 1 water : Discide solution (Alimed; Dedham, Massachusetts, USA) for twenty minutes. The components were scrubbed with a soft nylon brush to remove loose debris and soaked again in 10 : 1 water : Discide solution for twenty minutes. The femoral heads were then placed in an ultrasonication bath for two twenty-minute sessions to remove loose debris. Following cleaning, the femoral heads were air-dried and stored in air until inspection was performed.

### 2.2. Visual Scoring of Metal Transfer

The extent of metal transfer on the femoral heads was scored using a 3-point semiquantitative scale adapted from Kim et al. [1] and a diffused lighting technique established by Heiner et al. [10]. Three independent observers (EKF, JSJ, and JTS) scored each femoral head, discussing dissimilar visual scores until a consensus was reached. Resting on the taper surface, the heads were viewed while being shrouded in an opaque tube. The tube diffused the room lighting, replacing any reflections with a smooth white background. Only the portion of the head that would articulate with the acetabular liner in a normal gait pattern was observed and scored in this manner (i.e., the upper hemisphere) [6, 11]. A score of 1 was given when no metal transfer was observed. A score of 2 was given when minimal metal transfer was observed, defined as isolated marks, a few marks in concentrated areas, or markings very light in color. A score of 3 was given for severe metal transfer, that is, when a longitudinal, concentrated metal transfer stripe was observed or when metal transfer of any type was observed over the majority of the upper hemisphere [1]. We used the visual score of metal transfer as a screening process to identify femoral heads suitable for quantitative analysis.

### 2.3. Image Acquisition

Femoral heads were photodocumented using the same diffused lighting technique developed by Heiner et al. [10]. Each femoral head was wiped with water and isopropyl alcohol to remove any dust that accumulated during storage and then positioned above a dark blue background in the opaque tube to remove background reflection artifacts and diffuse the external lighting from the photography lights, respectively. The upper hemisphere of each femoral head was photographed using a Nikon D800 digital SLR camera (Nikon Inc.; Melville, New York, USA) mounted on an overhead stand and remote accessed from a computer. In order to increase our depth of field and to obtain an in-focus image of the entire upper hemisphere, we utilized focus-bracketing using commercial software (Helicon Focus 6, Kharkov, Ukraine). Ten images per femoral head were captured at different equidistant focal points and digitally stacked to achieve an extended depth of field.

### 2.4. Metal Transfer Surface Area Calculations

**Figure 1:**
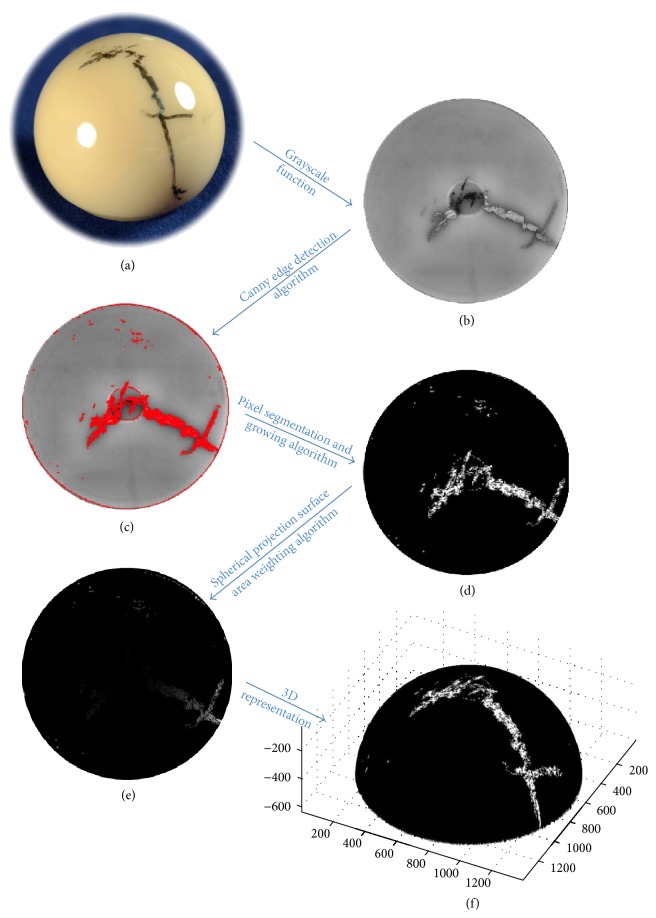
Procedure for analyzing metal transfer damage on a ceramic femoral head. (a) Initial photo documentation of a ceramic femoral head with metal transfer damage. (b) Photo documentation with grayscale enhancement applied. (c) Canny edge detection defining the edges of the metal transfer. (d) Pixel segmentation isolating metal transfer from the femoral head for the upper hemisphere, expanded to full metal transfer surface area using a Nearest-Neighbor growing algorithm. (e) Reverse azimuthal projection producing a weighted two-dimensional image of the total upper hemisphere featuring metal transfer, and the percent coverage. (f) An equivalent 3D representation of the isolated metal transfer.

We quantitatively analyzed components with visual evidence of metal transfer (corresponding to a transfer score ≥ 2), using a customized MATLAB (Mathworks; Natick, Massachusetts, USA) algorithm applied to all images of the femoral heads. All photographs were enhanced using a standard MATLAB monochromatic photograph filter. Metal transfer was identified using an edge detection filter (Canny Edge Detection) to isolate metal transfer using pixel segmentation [6, 10, 12]. Outlines of the transfer were filled in using a customized Nearest-Neighbor algorithm. The two-dimensional image was projected onto an idealized half-sphere using a reverse azimuthal projection (Figure 1). Metal transfer was reported as a percentage of the surface area of the upper hemisphere covered by metal transfer and then converted to the surface area in mm^2^ for a half sphere based on the femoral head size. A repeatability study was conducted in accordance with ASTM International Designation: E 691-08 [13]. It was determined that this procedure, when repeated three times consecutively under similar conditions by the same operator, allowed for an average repeatability standard deviation (*s*_*r*_) of 0.348% across the cohorts. This was derived from the standard deviation of the percentages produced three consecutive times per head, over an entire population of 74 analyzed heads ($s_{r}=\sqrt{\sum_{1}^{p}{(s}^{2}/p)};\;\;p=74$).

### 2.5. Pattern Classification

The upper hemispheres of femoral heads with evidence of metal transfer (transfer score ≥ 2) were inspected to determine different patterns of transfer through visual examination and digital microscopy (Keyence; Itasca, Illinois, USA). Seven pattern categories were discerned by three independent observers (EKF, JSJ, and JTS): solid patch, directional scratches, longitudinal stripe, random stripe, random patches, patterned coverage, and miscellaneous (Table 2). In the case of discrepancies between the observers, the differences were discussed until consensus was obtained.

**Figure 2:**
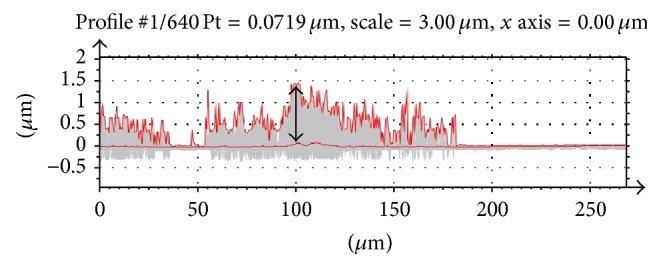
Optical microscopy surface scan converted into a series of profiles with profile envelopes for the maximum and mean values. Distance between the mean line and the highest peak was measured per scan and averaged per head (SR_pm_).

The dimensions of the three most frequently observed patterns (longitudinal stripe, random stripe, and random patches) were measured. The most prominent mark (i.e., largest metal transfer surface area, darkest, and most consistent in color) was measured for total length and width using calibrated calipers. Length was considered to be the main exhibited direction, and each mark was measured only to the upper hemisphere boundary at the head equator. Arc length was calculated from the chord length measured with the calipers (Arc  Length = Diameter*∗*sin^−1^⁡(Measured  Chord  Length/Diameter)). Metal transfer height was measured using white light interferometry (WLI) (NewView 5000, Zygo; Middlefield, Connecticut, USA). A total of 5 WLI measurements per femoral head were taken for visually identified regions of metal transfer. Using commercial three-dimensional surface analysis software (TalyMap Platinum, Taylor Hobson, Leicester, United Kingdom), the spherical form was removed from each surface scan and converted into a series of two-dimensional height profiles encompassing the entire measured surface (Figure 2). The maximum metal transfer peak height for each femoral head was calculated as the mean peak areal height (SR_pm_): the single maximum mean-to-peak height per each measurement location averaged over all scanned regions (Figure 2).

### 2.6. Statistical Analysis

Nonparametric statistical analysis was performed using commercially available software (SPSS Statistics 22; Chicago, Illinois) due to the nonnormal nature of the data. To determine differences between bearing couples, we used the Kruskal Wallis analysis of variance with* post hoc* Dunn's test, where appropriate. For correlations between continuous variables, Spearman's Rank Correlation test was used. Alpha was set to 0.05 for all tests. All results are reported as median, IQR.

## 3. Results

**Figure 3:**
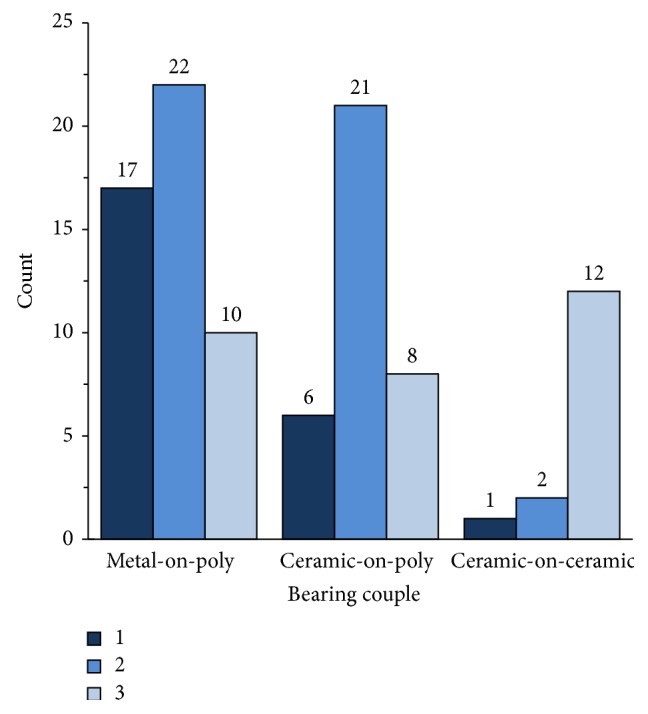
Count of femoral heads in each material cohort (M-PE, C-PE, and C-C) belonging to each visual score classification. Scores ≥ 2 were analyzed further for having visual metal transfer.

Metal transfer was a common observation for all bearing couples; however, it occurred in different proportions among the three cohorts (*p* < 0.001; Pearson Test). Metal transfer (transfer score ≥ 2) on the upper hemisphere was observed in 64% (*n* = 32/50) of M-PE heads, 83% (*n* = 29/35) of C-PE heads, and 93% (*n* = 14/15) of C-C heads (Figure 3). Altogether, 75 of 100 femoral heads had a metal transfer score ≥ 2. Within these 75 femoral heads, severe or concentrated metal transfer (transfer score = 3) was observed on 20% (*n* = 10/50) of M-PE heads, 23% (*n* = 8/35) of C-PE heads, and 80% (*n* = 12/15) of C-C heads.

**Figure 4:**
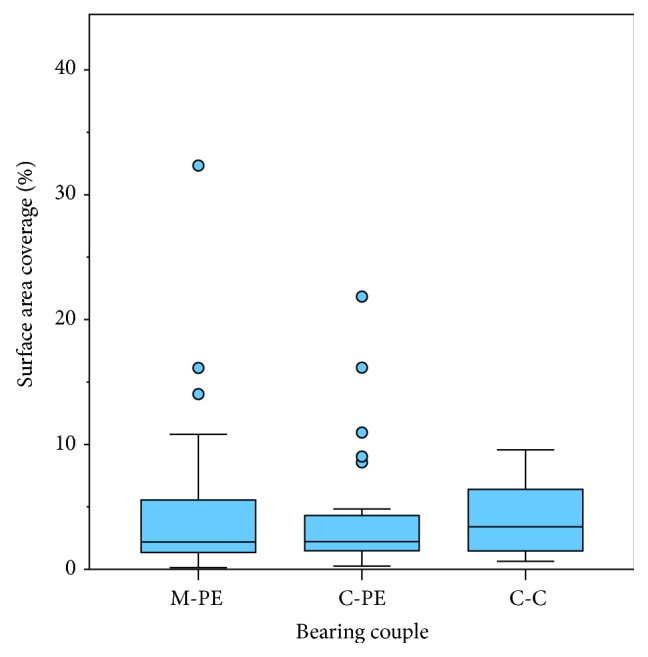
The distribution of metal transfer surface coverage was similar across the three material cohorts (*p* = 0.90).

For the 75 femoral heads that exhibited metal transfer with a visual score ≥ 2, the metal transfer surface area coverage was similar among the 3 cohorts, with an overall median coverage of 2.3% of the upper hemisphere (IQR: 4.1%; *p* = 0.90) (Figure 4). The median metal transfer surface area coverage was 2.2% (IQR: 4.3%) for the M-PE cohort, 2.2% (IQR: 2.9%) for the C-PE cohort, and 3.4% (IQR: 5.3%) for the C-C cohort. Similarly, the curved surface area of metal transfer was similar among the three cohorts (overall median surface area = 39.8 mm^2^ (IQR: 67.5 mm^2^); *p* = 0.98). The surface area of metal transfer was 38.6 mm^2^ (IQR: 70.9 mm^2^) for the M-PE cohort, 35.7 mm^2^ (IQR: 50.0 mm^2^) for the C-PE cohort, and 54.7 mm^2^ (IQR: 99.7 mm^2^) for the C-C cohort. There was no difference in metal transfer surface area coverage between ZTA and alumina heads in the C-PE cohort (*p* = 0.27) or between conventional and HXLPE for the M-PE and C-PE cohorts (*p* = 0.44, *p* = 0.53, resp.). Patient weight, implantation time, BMI, head size, age, and gender were not correlated with the metal transfer surface area coverage in any of the cohorts (*p* > 0.16).

**Figure 5:**
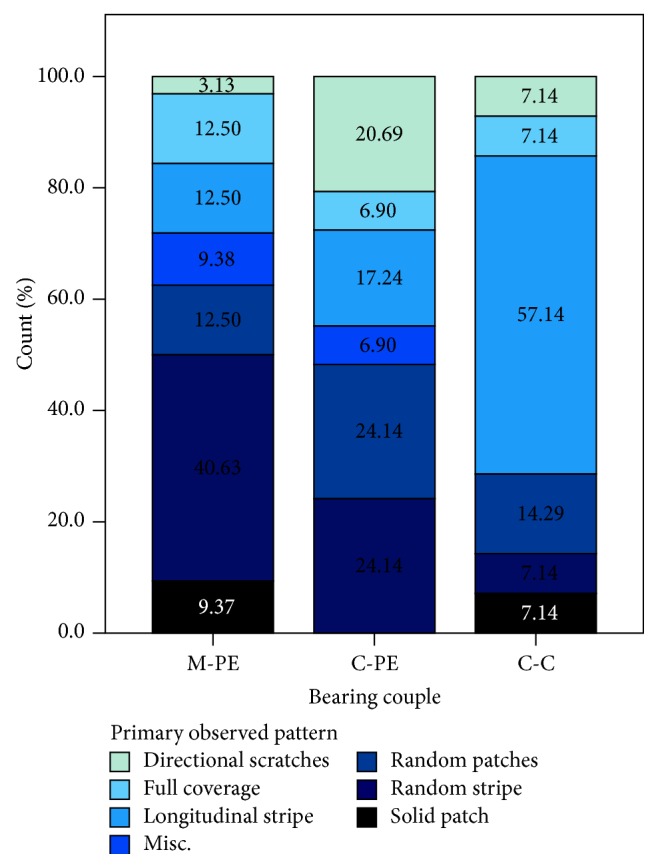
Distribution of identified metal transfer patterns across the three cohorts.

Seven metal transfer patterns were observed on the 75 analyzed femoral heads across all three cohorts, occurring in different proportions (*p* = 0.02; Figure 5).

**Figure 6:**
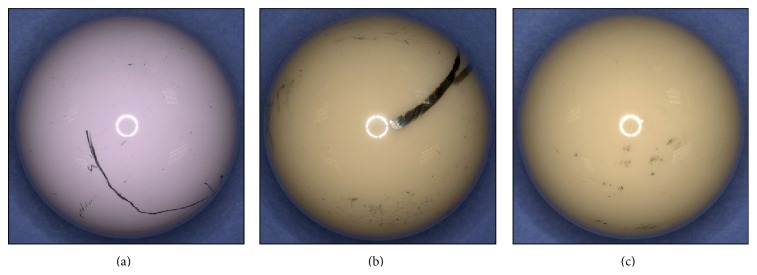
Digital photographs of the three most common patterns: (a) random stripe, (b) longitudinal stripe, and (c) random patches. Both the longitudinal stripe and the random stripe also feature the most common secondary pattern: patterned coverage over the entire upper hemisphere.

For femoral heads with evidence of metal transfer, the three most common primary patterns observed were random stripe (*n* = 21/75), longitudinal stripe (*n* = 17/75), and random patches (*n* = 13/75) (Figure 6). For 72% (*n* = 54/75) of the femoral heads, only one of the seven metal transfer patterns was observed on the upper hemisphere. A secondary metal transfer pattern (lighter in color, less surface area coverage, and more sporadic in appearance) was observed for 28% (*n* = 21/75) of the femoral heads: 12% (*n* = 9/75) of the M-PE cohort; 9% (*n* = 7/75) of the C-PE cohort; and 7% (*n* = 5/75) of the C-C cohort. The most common secondary pattern observed was patterned coverage (*n* = 6/21) accompanying a longitudinal stripe (*n* = 5/21).

Although the surface area coverage of the upper hemisphere was similar among the cohorts, we did observe differences in morphology of the three most prevalent primary patterns (Table 3). In particular, the arc length of the predominant pattern was shorter in the M-PE cohort than both the C-PE cohort and the C-C cohort (mean difference = 5.7 mm and 7.11 mm; *p* = 0.006, *p* = 0.001, resp.). In addition, the arc width was also smaller for the M-PE cohort compared to the C-C cohort (mean difference = 0.78 mm, *p* = 0.001), and the height of the metal transfer was significantly taller in the M-PE cohort than the C-C cohort (mean difference = 0.84 mm, *p* = 0.022). With the numbers available, we could not detect a difference in arc length, arc width, or metal transfer height between the two cohorts with ceramic heads (*p* = 0.805, *p* = 0.138 and 1.000 for the arc length, arc width, and transfer height, resp.).

## 4. Discussion

Metal transfer has been observed on retrieved femoral heads in total hip replacements.* In vitro* studies have observed increased wear of the polyethylene acetabular liner with the presence of metal transfer [8]. Although this has been observed in retrieved components, little is known about the effect (if any) of the bearing surface material on the morphology of the metal transfer. This study investigated whether observed metal transfer was more prominent on one bearing surface couple over another, and if metal transfer on these surfaces had a common morphology. We found that metal transfer was a common observation on the femoral heads for the bearing surface couples investigated in this study. Moreover, for heads that did have evidence of metal transfer, we found that amount of surface area covered by metal transfer was similar among the M-PE, C-PE, and C-C cohorts. However, observed metal transfer was greater in height and shorter in length on CoCr compared to ceramic femoral heads. Clinical factors (weight, BMI, implantation time, etc.) were not predictors of the amount of metal transfer observed on the upper hemisphere.

There were limitations to this study. We only measured damage on the upper hemisphere. Although we did observe damage on the heads outside of this area, these marks were excluded from our analysis in order to only capture transfer that would likely have an impact on the bearing surface of the acetabular liner during normal use. This study only addressed revised hip replacements, and thus the findings may not be reflective of functioning implants that are* in vivo*. We did not consider if femoral heads articulated in extreme positions, such as acetabular cups with a highly vertical placement, which could lead to a very different articulating zone. The opposing articulating surface was also not examined, which could give additional information about causation mechanisms. Although metal transfer has been observed in unstable implants that have undergone dislocations and closed reductions, only one implant in this study was revised for instability. According to a community registry study performed in the past year on 6,801 revision cases over twenty years, instability/dislocation was the cause of failure for 1.7% of total hip replacements [14]. The cohorts in the current study likely underrepresent components revised for instability, making it likely that this study underestimated the amount of metal transfer across our retrieval collection of revised hips. Also, the metal transfer was visually more apparent in the ceramic cohort when compared to the CoCr cohort. Although the diffuse lighting technique made by Heiner et al. [10] for viewing CoCr femoral heads helped define the areas of metal transfer, it is possible that we underestimated the amount of transfer in the CoCr cohort due to the similarity in color.

The original CoCr and ceramic femoral head cohorts were matched for flexural rigidity of the implant stem, with no consideration of bearing surface characteristics. This ensured a random sampling of femoral heads when examining the bearing surface. To the author's knowledge, this is the largest sample size to date to be examined for metal transfer on the bearing surface, the next largest sample size being 27 explanted ZTA femoral heads studied by Elpers et al. [11]. Additionally, the height profiles for each WLI measurement were cumulative of the entire scanned region (365 linear profiles for both the longitudinal and lateral directions), rather than a representative orthogonal profile. Using SR_pm_ was an advantage over commonly used roughness parameters (Sa, Ra) that average both material loss (depth) and material gain (height) from the bearing surface center line [1, 5, 8]. SR_pm_ provided the height of just the metal transfer above the bearing surface center line (representing the unworn femoral head), achieving more accurate height dimensions. Furthermore, batch processing performed in MATLAB on the profiles and photographs allowed for high throughput on a large cohort.

The results reported here are consistent with the work of past reports that identified metal transfer on the bearing surface of CoCr and ceramic femoral heads. In the Elpers et al. [11] study of ZTA femoral heads, metal transfer was observed on 60% of the femoral heads at the apex and 95.6% of the femoral heads at the equator. Similarly, the current study observed metal transfer on 75% of the femoral heads examined, over the region of apex to equator. The surface area percentages reported here are also similar to the work of Kim et al. [1], where they observed areas identified as metallic-like “smearing” covering 1 to 10% of the total head surface in a study of 15 retrieved ceramic heads. An additional retrieval study performed by Müller et al. [5] found metal transfer surface areas on the total bearing surface of 5 mm² to 8 mm² per metal transfer mark. In this study the results are generally an order of magnitude higher than the other studies. This may be because we considered the entire curved surface area of the upper hemisphere. It is unclear how the previous studies calculated the surface area, or if they accounted for the spherical form in the calculation. With the numbers available, we were unable to detect a difference in metal transfer surface area coverage among the cohorts. However, the implications of this finding on the clinical performance of ceramic femoral heads (particularly wear) are unknown at this point. In a hip simulator study of zirconia and CoCr heads on conventional polyethylene disks, Eberhardt et al. [8] reported higher levels of polyethylene wear for both materials that had induced transfer when compared to undamaged femoral heads. However, the femoral heads with induced transfer showed a postsimulation positive skewness (*R*_sk_) more than twice that of the femoral head retrievals in their study. On the bearing surface of a femoral head, a positive skewness suggests material build-up whereas a negative skewness suggests material loss. The high positive values reported suggest that the induced metal transfer height for simulation did not replicate the induced metal transfer height* in vivo*, warranting simulator studies with more accurate generation of metal transfer morphology. In addition, Kim et al. [1] found in a retrieval study that femoral heads with severe “smearing” had significantly higher polyethylene wear rates than femoral heads with slight “smearing” on conventional polyethylene. Thus, a future retrieval study documenting conventional and HXLPE wear in the context of metal transfer surface area on the femoral head is warranted.

Of the seven distinct patterns identified within the three cohorts, random stripes and random patches were the most common for both the M-PE and C-PE cohorts. These are hypothesized to be from 3rd body debris trapped in the articulating space, such as particles of porous coatings on acetabular shells, or material removed from impingement of the shell and neck [8]. These markings are also consistent with those noted in other reports, although in most reports there is no form of pattern classification system presented except for the six case studies performed by Heiner et al. [7, 11]. Previous studies suggest that the most common cause of metal transfer is impingement and dislocation, exhibiting broad regions of micro and macro scraping and longitudinal directionality (here represented by the longitudinal stripe pattern) [6, 7]. In the current study, 23% (*n* = 17/75) of the patterns identified had longitudinal stripes. Most of the previous work investigating metal transfer was performed on selected cohorts from patients who were unstable or had multiple dislocations [1, 8, 9]. Therefore, the differences may be a result of selection bias. In this study the longitudinal stripe pattern was less common for the M-PE cohort (13%, *n* = 4/32) than the C-PE and C-C cohorts (26%, *n* = 9/35; 44%, *n* = 4/9, resp.). In previous literature, longitudinal stripes on ceramic bearings have been attributed to direct contact between the raised Ti acetabular shell rim and the femoral head during dislocation or the closed reduction process [5, 7]. However, only 4 out of 13 ceramic heads with a longitudinal stripe pattern of transfer had a ceramic liner with a raised Ti edge. The longitudinal stripe height was greater for the M-PE cohort compared to both of the ceramic cohorts. This height difference could be attributed to the hardness of modern-day ceramic femoral heads, resulting in the softer transferred metal to be worn down by the normal gait cycle more easily than on CoCr femoral heads [5, 15]. Nevertheless, the mean height differences observed between the M-PE and the two ceramic cohorts (C-PE and C-C) were less than one micron. Therefore, the clinical impact of these observed differences remains unclear.

Miscellaneous and unusual metal transfer patterns that did not fall into the seven pattern groups were noted on two ceramic and four CoCr femoral heads of the 75 analyzed. Both of the ceramic heads had extreme metal transfer. One head showed tooling marks originating at the taper and extending towards the apex of the femoral head, indicative of iatrogenic damage. The other ceramic femoral head had extensive metal transfer occurring both on the unworn portion (appearing shiny) and significantly worn portion (appearing dull and rough to the touch) of the bearing surface [16]. This head had completely worn through the polyethylene liner and had been articulating solely against the Ti alloy acetabular shell, resulting in gross amounts of Ti transfer to the bearing surface. Two of the miscellaneous CoCr heads showed varying degrees of brown coloring with a cloudy surface finish. Although the cause of this is unclear, the pattern was similar to the circumferential discoloration pattern observed by Heiner et al. from Ti corrosion, albeit to a lesser degree. The two other CoCr heads showed highly roughened areas similar to the visual characteristics noted by Heiner at al. [7] as pitting underneath metal transfer deposits, possibly causing the original deposits to detach from the bearing surface.

## 5. Conclusion

This retrieval study compared metal transfer presence on CoCr and ceramic femoral heads and established a categorization method to describe the morphology of the common forms of observed metal transfer. Understanding the morphology of metal transfer may be useful for more accurate polyethylene wear studies through more realistic recreation of metal transfer in* in vitro* pin-on-disk and joint simulators studies. This would allow for metal transfer and its correlation to polyethylene wear to be more accurately studied under normal and adverse activities and for predictive wear studies of failure mechanisms to be performed on HXLPE.

## Figures and Tables

**Table 1:** Patient demographics were similar between the three cohorts with the exception of patient age. All data is reported as median, IQR, with the exceptions of gender (reported as percent female).

	CoCr-on-polyethylene femoral heads	Ceramic-on-polyethylene femoral heads	Ceramic-on-ceramic femoral heads	*p* value
Age (years)	57 (16.0)	53 (15.3)	49 (11.4)	0.02
Gender (% female)	50%	37%	27%	0.21
Body mass index (kg/m^2^)	29.1 (11.7)	28.2 (11.9)	30.7 (7.3)	0.54
Weight (kg)	185 (71.5)	189 (75.0)	200 (82.8)	0.37
Head size [median] (mm)	32 (8.0)	32 (4.0)	32 (0.0)	0.47
Max UCLA activity score	6 (4)	6 (3)	6 (3)	0.64
Implantation time (years)	1.9 (2.7)	2.1 (4.4)	1.7 (1.4)	0.77

**Table 2:** Summarized pattern categories of observed metal transfer on the bearing surface of the analyzed femoral heads. Patterns are presented by type, description, and an exemplar photograph for each cohort (images taken with a digital microscope (Keyence; Itasca, Illinois, USA)).

Pattern observed	Description	CoCr	Ceramic
Solid patch	Similar in length and width, with a rough dark gray appearance. Often only one per femoral head, and commonly the only metal transfer on the bearing surface. Occasionally accompanied by one or two random patches	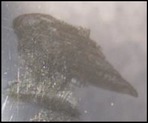	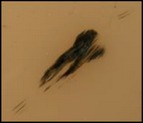

Directional scratches	Multiple thin lines of transfer with similar macrodirectionality. Typically clustered in groups or in a circular ring around the apex of the femoral head	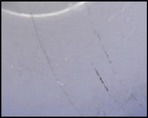	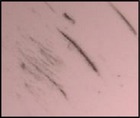

Longitudinal stripe	Longitudinal dark marks appearing black on ceramic and dark gray, brown, or dull gray on CoCr. Often extending from taper to apex of the femoral head with strong macrodirectionality, opposing microdirectionality, and one or both longitudinal edges straight and well defined. Often only one mark of this type per head, accompanied by additional transfer patterns (see random patches, patterned coverage)	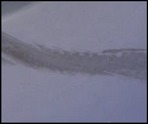	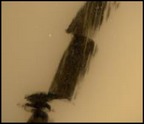

Random stripe	Similar coloring to a longitudinal stripe, with a high length : width ratio and no preferred orientation (lateral or longitudinal). It can be straight, curved, or looped; one to two seen per upper hemisphere	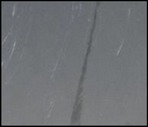	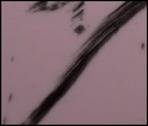

Random patches	No overall directionality, often overlapping marks. Found in clustered groups or independently with no location preference. Typically one to a few marks, either the only pattern observed or a secondary pattern	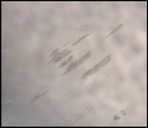	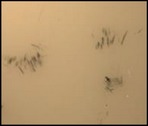

Patterned coverage	Small straight lines or pinpoints markings, evenly distributed over the entire upper hemisphere. Spaced approximately 1 mm apart in every direction. Most often a secondary pattern to a longitudinal transfer stripe	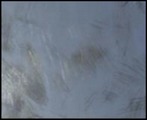	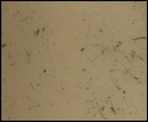

Miscellaneous	Iatrogenic damage, stripe wear accompanied by metal transfer, additional surface damage, or unconfirmed metal transfer	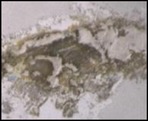	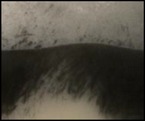

**Table 3:** Dimensions of the most prominent mark for the three most prevalent primary transfer patterns. Sample size was determined by the amount of femoral heads per cohort exhibiting each pattern (longitudinal stripe, random stripe, and random patches, resp.). Data is reported as the mean ± standard deviation.

	M-PE			C-PE			C-C		
	Length (mm)	Width (mm)	Height (*µ*m)^*∗*^	Length (mm)	Width (mm)	Height (*µ*m)	Length (mm)	Width (mm)	Height (*µ*m)^*∗*^
Longitudinal stripe	12.3 (11.7)	1.9 (1.3)	1.2^*∗*^	9.1 (10.3)	1.5 (0.9)	0.5 (0.2)	16.2 (5.0)	2.2 (1.7)	0.4 (0.6)
Random stripe	7.3 (5.0)	0.7 (0.5)	1.8 (1.9)	14.5 (2.9)	1.2 (1.9)	1.0 (1.1)	5.9^*∗*^	1.2^*∗*^	2.3^*∗*^
Random patches	2.6 (2.1)	1.2 (1.0)	1.9^*∗*^	3.6 (3.8)	1.2 (2.9)	0.7 (0.9)	6.2^*∗*^	3.1^*∗*^	0.9^*∗*^

^*∗*^Height only available for 10 C-C heads and 13 M-PE heads, with insufficient data to present and interquartile range in some cases.
